# Supplementary material for: Quantifying the Risk and Cost of Active Monitoring for Infectious Diseases
Source: Sci Rep. 2018 Jan 18;8:1093. doi: 10.1038/s41598-018-19406-x (PMC5773605; doi:10.1038/s41598-018-19406-x)
Supplement: Supplementary file 1 — Supplementary Information [file 41598_2018_19406_MOESM1_ESM.pdf]

Methodological supplement for: Quantifying the Risk and Cost of Active Monitoring for Infectious Diseases

Nicholas G Reich, Justin Lessler, Jay K Varma, Neil M Vora*

September 2017

Estimation of incubation period

All incubation period observations were treated as single interval-censored data, which means that intervals of possible incubation periods were observed rather than exact values (1). The data used for these analyses are publicly available at https://github.com/reichlab/activemonitr (current version of code), or at https://doi.org/10.5281/zenodo.260135 (static release as of 26 January 2017).

We fitted the observed data for each disease to a gamma probability distribution using Bayesian Markov Chain Monte Carlo (MCMC) methods with the Metropolis-Hastings algorithm. The gamma distribution is one of several "heavy-tailed" distributions often used to describe incubation periods, and aligns with assumptions made by previous researchers. (2) We assumed uninformative, flat priors on the shape and scale parameters of the gamma distribution.

We used a standard procedure to evaluate MCMC convergence for each of the pathogens. First, we adjusted the size of the proposal distributions to obtain acceptance rates between 30% and 40%. Second, we visually inspected the resulting chains to assess convergence. Third, we thinned the posterior samples to reduce auto-correlation between observations. Fourth, we computed the Gelman and Rubin $\hat{R}$ multiple sequence diagnostic to assess convergence across chains (3). Fifth, we specified the appropriate number of samples, burn-in, and thinning rates to obtain 1 million posterior samples for smallpox and MERS-CoV and 3 million for Ebola.

For smallpox, we ran 20 parallel MCMC chains each with 320,000 samples. We discarded the first 20,000 iterations as burn-in and removed every sixth sample. This procedure yielded 1 million samples from the posterior distribution.

For MERS-CoV, we ran 20 parallel MCMC chains each with 120,000 samples, discarding the first 20,000 iterations as burn-in and thinned every other sample. This procedure yielded 1 million samples from the

posterior distribution.

For Ebola, the data structure was different. Rather than having a unique single interval censored observation for every case, we had 300 draws from the posterior distribtion of incubation periods for each individual in the transmission tree. We ran a single parallel MCMC chain for each of the 300 posterior samples. Each chain had 110,000 samples, we discarded the first 100,000 as burn-in and did not thin. This procedure yielded 3 million samples from the posterior distribution. We chose to retain more samples for this analysis to account for the greater number of chains being run.

Two-dimensional credible regions were estimated using kernel density estimation on the joint posterior distribution of gamma parameters with flexible bandwidth matrices estimated by multivariate smooth cross-validation.(4,5)

Missing data had limited impact on our analysis because reported cases typically have known possible times of infection. There were no missing data for either MERS or smallpox. Seven index cases out of 152 total cases from the Ebola outbreak in Guinea did not have known possible times of infection. Incubation periods were not estimated for these individuals in the original manuscript describing this outbreak(6), and these seven observations were excluded from our analysis, leaving 145 observations of the incubation period for Ebola. We note that there may be biased sampling of incubation periods as typically only more severe cases may be reported or observed, and these cases may have different incubation periods than cases with less severe symptoms.

Estimates of probabilities of infection

To provide first approximations of the probabilities of infection associated with each exposure risk category, we used available data on cases diagnosed in the United States (as the numerator) and number of individuals monitored in the United States (as the denominator). Because no cases were diagnosed in the United States after monitoring programs were implemented, we extrapolated the number of monitored individuals to cover the entire duration of the outbreak.

We used public data on the four cases diagnosed in the United States to classify these cases into appropriate exposure-risk categories.(7,8) Although some news reports have indicated that the Dallas index case may have had direct exposure to infected individuals, reports of initial statements made by the index case do not cite this known exposure, therefore he would have likely been classified as being at low (but not zero) risk. The two Dallas healthcare workers, per CDC exposure risk definitions, would also have been considered low

(but not zero) risk.(9) The New York City case would have been classified as some risk, due to his recent work in an Ebola treatment center abroad.(10)

Supplemental Table 1: List of known Ebola cases diagnosed in the U.S.

case	date of symptom onset	exposure risk category
Dallas index case	Sept 25, 2014	low (but not zero)
Dallas healthcare worker 1	Oct 11, 2014	low (but not zero)
Dallas healthcare worker 2	Oct 11, 2014	low (but not zero)
NYC doctor	Oct 23, 2014	some risk

Stehling-Ariza et al.(11) reported 10039 monitored low but not zero risk individuals and 315 some or high risk individuals over a four month period (November 3, 2014 through March 8, 2015). Based on World Health Organization case report data downloaded from the CDC website (12), we estimate that 39% of all reported Ebola cases in Guinea, Liberia, and Sierra Leone through February 2016 occurred during this four month period.

From these numbers, we extrapolate that 10039/ 0.39 or roughly 26000 individuals at low (but not zero) risk could have been eligible for monitoring over the course of the entire outbreak. Similarly, we extrapolate that 1000 some or high risk individuals could have been eligible for monitoring in the United States over the entire course of the outbreak. Both extrapolations have been rounded to the nearest thousand.

Supplemental Table 2: Relevant data for estimating the probabilities of developing Ebola for each CDC exposure-risk level.

CDC risk level	Number of cases reported in the US	Estimated denominator for whole outbreak	Estimated probability of developing Ebola (ϕ)
"Low (but not zero) risk"	3	26,000	1/10,000
"Some risk or high risk"	1	1,000	1/1,000

Estimates of probability of developing an unrelated infection

There were 5,379 non-unique persons who underwent active monitoring by DOHMH during October 25, 2014 to November 7, 2015. This represented 5,025 unique persons, some of whom were monitored on more than

one occasion. During the course of these individuals being actively monitored by DOHMH, some individuals developed symptoms that could possibly have indicated the onset of Ebola. Specifically, there were 122 occasions of an individual experiencing fever, diarrhea, vomiting, or unexplained bleeding/bruising. These symptomatic individuals were evaluated by DOHMH staff, and 39 were referred for follow-up testing for Ebola at a hospital or other medical facility. We assumed that 30 of those seeking care sought care at a hospital, representing 1% of the total number of monitored individuals. Subsequent analyses showed little sensitivity to this assumption, as the overall proportion was so small. Specifically, for the Ebola case study, increasing the hospitalization rate by a factor of 15 decreased by 1 day the duration of active monitoring that minimizes the expected cost.

Our aim was to develop a simple model for the rate at which individuals were falling ill from causes other than Ebola. To do this, we performed a simple discrete-time survival analysis, assuming that the hazard of developing symptoms from other sources was constant over time. We observed a single aggregated datapoint, that after 21 days of monitoring, only a small fraction of individuals had reported symptoms that required hospitalization: 30 / 5379 = 0.0056. Assuming that every day during a monitoring period had the same risk of developing symptoms, we can write the survival function, the probability that an individual would be symptom free after t days, as

$$S(t) = (1 - \lambda)^t$$

By plugging in $S(21)$ = 1- 0.0056, we solve for λ, the constant per-day hazard, and obtain the estimate $\hat{\lambda}$ = 0.27/1000. This translates into one hospitalized case per every 3755 person-days of active monitoring. Therefore, for the model below we estimate the probability of a monitored individual developing Ebola-like symptoms during a d day monitoring period as $r_d = 1 - (1 - \hat{\lambda})^d$.

Probabilistic model

We developed a probabilistic model for evaluating the likelihood that a monitored individual would develop symptoms at different stages of monitoring. This model is based on five key parameters: the probability of a monitored individual developing symptomatic infection (ϕ), two parameters that define the distribution of incubation periods (T denotes the random variable for incubation periods), probability of a monitored individual developing symptoms like the disease of interest during a d day monitoring period (r_d from above), and either a fixed or random duration of time between the time of infecting exposure and the onset of active monitoring (u).

The model-based probabilities for the disease of concern (see Figure 1, main text) are shown in Table 3.

Supplemental Table 3: Outcomes and associated probabilities for model

outcome	probability
does not develop disease of concern	$p_1 = 1 - \phi$
does not develop disease of concern & not hospitalized for other symptoms	$p_1(1 - r_d)$
does not develop disease of concern & hospitalized for other symptoms	$p_1 r_d$
develops disease of concern during active monitoring	$p_2 = \phi \cdot Pr(T \leq d + u)$
develops disease of concern after active monitoring	$p_3 = \phi \cdot Pr(T > d + u)$

We quantified the uncertainty about the probability for each outcome scenario attributable to parameter uncertainty (in our estimates of the incubation period distribution) and to uncertainty associated with not knowing the time between infection and the beginning of active monitoring. For parameter uncertainty, we computed each outcome scenario probability from a sample of the posterior distribution of gamma parameters for a fixed u. For uncertainty in the infection time, we assumed that u was distributed uniformly, and calculated outcome probabilities for a set of random samples from this distribution while holding the gamma parameters fixed at their posterior median values. To account for both uncertainties, we sampled simultaneously from both distributions.

We chose the maximum u value such that $max(u)/2 + m = 90^{th}$ percentile of T. This implies that for Ebola $max(u) = 16.8$ days.

While our model explicitly propagates uncertainty in the incubation period distribution, a formal, data-driven understanding of the uncertainty for other model parameters is not likely to be available to public health practitioners in practice. For one of the most critical model parameters, the probability that a monitored individual is infected with the disease of interest, our model can assess several different plausible values at a single time. Four model parameters (reproductive rate of the disease, cost of a false positive, cost per person-day of monitoring, and cost per case) are each specified to lie within a fixed range. A single parameter, the per-day risk of acquiring an unrelated infection, is assumed to be a single fixed value.

Sensitivity analyses: model assumptions about risk

We conducted sensitivity analyses to calculate the duration of active monitoring that minimized the maximum expected cost for a range of different probabilities of developing symptomatic disease. Assuming the same cost structure for active monitoring, we used our model to estimate a cost range of monitoring for each pathogen.

Table 4: Supplemental Table 4: Results of sensitivity analysis testing the impact of varying probabilities of symptomatic illness (phi) with the duration of active monitoring that minimizes the maximum expected cost.

disease	phi	optimal_duration
Ebola	1/10	48.0
Ebola	1/100	40.0
Ebola	1/1000	31.1
Ebola	1/10000	21.3
MERS-CoV	1/10	29.0
MERS-CoV	1/100	24.9
MERS-CoV	1/1000	20.0
MERS-CoV	1/10000	15.2
Smallpox	1/10	23.2
Smallpox	1/100	22.0
Smallpox	1/1000	19.5
Smallpox	1/10000	17.1

Sensitivity analysis: outlying Ebola incubation periods

We ran a sensitivity analysis on our incubation period estimation. We left out the two observations that had a mean incubation period (across the posterior estimates provided as raw data) of greater than or equal to 25 days. One observation had a mean incubation period of 34.4 days and another of 29.5 days. Leaving these observations out did not have a substantial impact on the estimated parameters. The estimated median incubation period decreased by about 0.2 days and the estimated 95th percentile of the incubation period

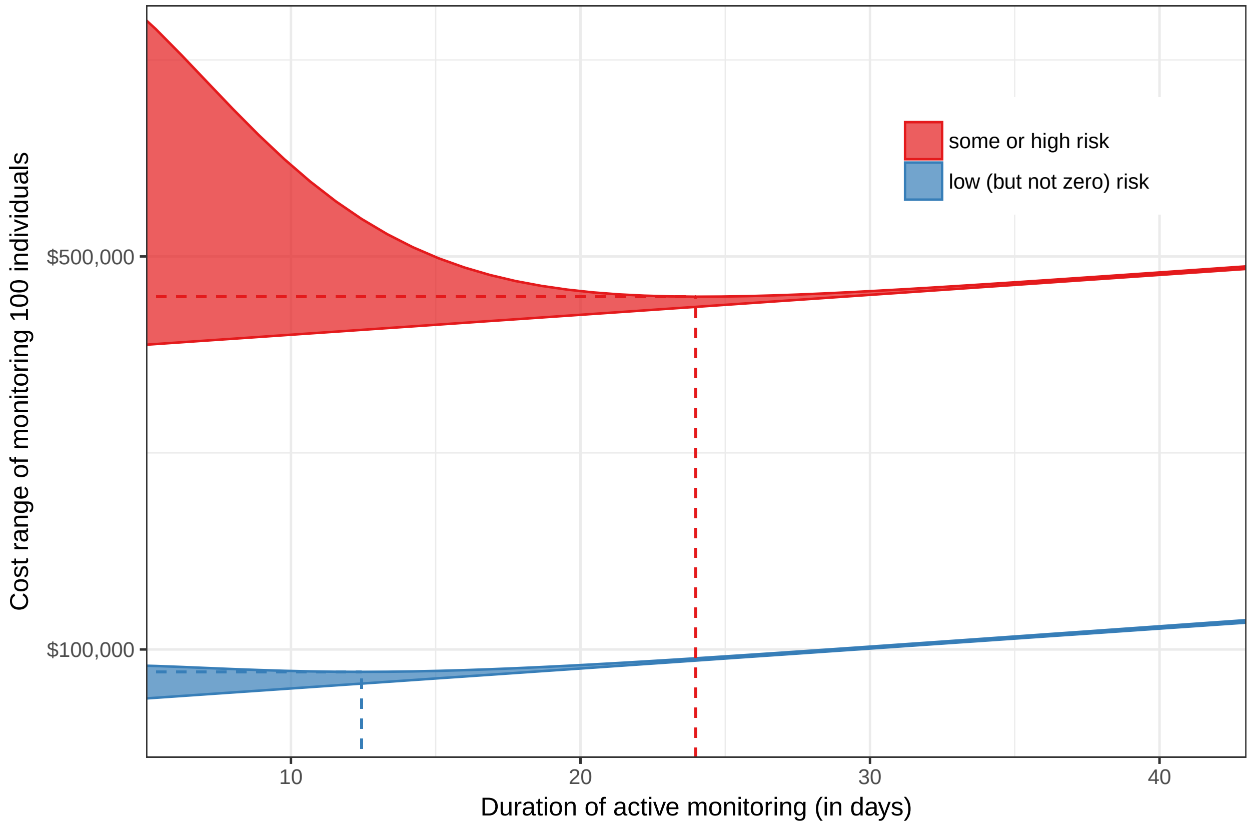

Figure 1: Estimated cost ranges of actively monitoring 100 individuals for Ebola, calculated separately for some or high risk individuals and low (but not zero) risk individuals. The dashed lines intersect at the minimum point for the upper limit of each cost range. This figure is a comparison to Figure 4 in the main text, but uses different parameters, specifically, it assumes that a case gives rise to at most one secondary infection, that the cost is a fixed $4 million per case, and that the cost per monitored-person day is $20

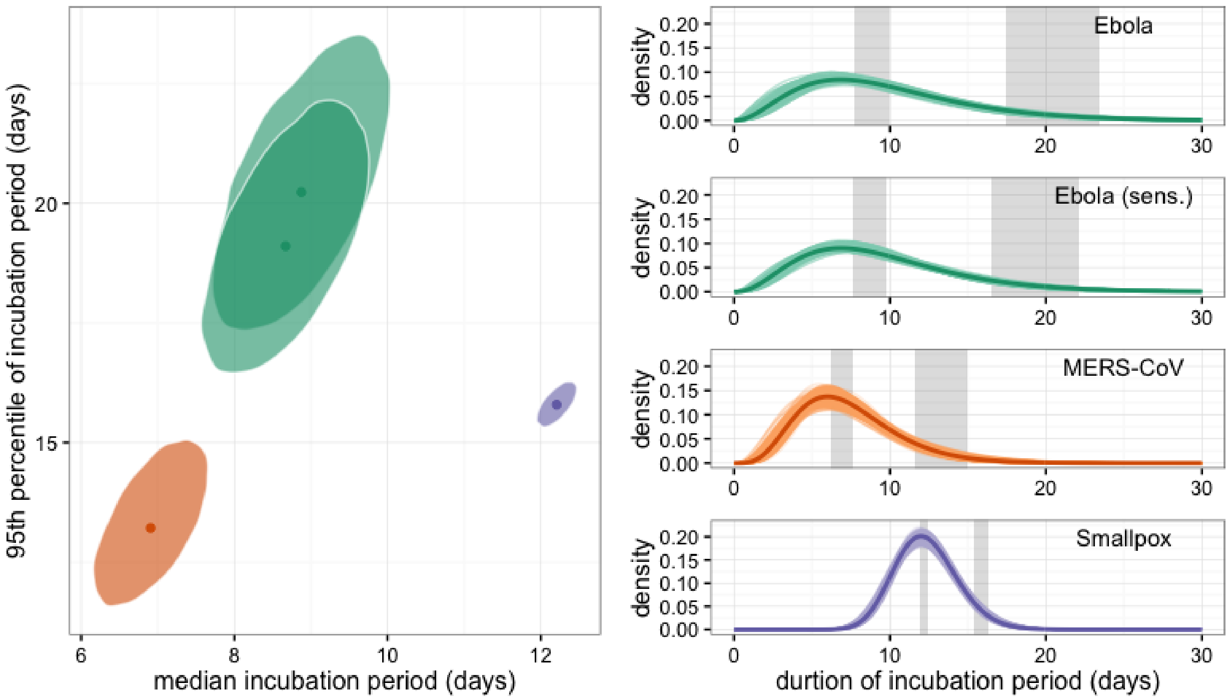

Figure 2: Estimates and credible regions for incubation period distributions for Ebola, MERS-CoV and smallpox. The second plot for Ebola shows the posterior distribution leaving out the two observations that had incubation periods above 25 days. The shaded elliptical areas represent regions that contain 95% of the estimated posterior distributions for each of the three diseases. The disease-specific curves plotted on the right show the estimated distribution for the incubation period for each disease (dark line). To show some of the uncertainty associated with these estimates, a random selection of density functions sampled from the joint posterior are represented by colored transparent lines around the heavy lines. Shaded vertical bands indicate the marginal credible regions for the median and 95th percentile.

decreased by 1 day (Figure 1).

Bibliography

1. Reich NG, Lessler J, Cummings DAT, Brookmeyer R. Estimating incubation period distributions with coarse data. Statistics in medicine. 2009 Sep;28(22):2769–84.

2. Virlogeux V, Park M, Wu JT, Cowling BJ. Association between Severity of MERS-CoV Infection and Incubation Period. Emerging Infectious Diseases. 2016;22(3).

3. Gelman A, Rubin DB. Inference from Iterative Simulation Using Multiple Sequences. Statistical Science. 1992;7(4):457–72.

4. Duong T, Hazelton ML. Cross-validation Bandwidth Matrices for Multivariate Kernel Density Estimation.

Scandinavian Journal of Statistics. 2005 Sep;32(3):485–506.

5. Duong T. ks: Kernel Smoothing. R package version 1.10.0. 2015.

6. Faye O, Boëlle P-Y, Heleze E, Faye O, Loucoubar C, Magassouba N, et al. Chains of transmission and control of Ebola virus disease in Conakry, Guinea, in 2014: an observational study. The Lancet Infectious Diseases. 2015 Mar;15(3):320–6.

7. Centers for Disease Control and Prevention. Cases of Ebola Diagnosed in the United States | Ebola Hemorrhagic Fever | CDC [Internet]. Available from: http://www.cdc.gov/vhf/ebola/outbreaks/2014-west-africa/united-states-imported-case.html

8. Centers for Disease Control and Prevention. Interim U.S. guidance for monitoring and movement of persons with potential Ebola virus exposure [Internet]. Available from: http://www.cdc.gov/vhf/ebola/exposure/monitoring-and-movement-of-persons-with-exposure.html

9. Chevalier MS, Chung W, Smith J, Weil LM, Hughes SM, Joyner SN, et al. Ebola virus disease cluster in the United States–Dallas County, Texas, 2014. MMWR Morbidity and mortality weekly report. 2014 Nov;63(46):1087–8.

10. Yacisin K, Balter S, Fine A, Weiss D, Ackelsberg J, Prezant D, et al. Ebola virus disease in a humanitarian aid worker - New York City, October 2014. MMWR Morbidity and mortality weekly report. 2015 Apr;64(12):321–3.

11. Stehling-Ariza T, Fisher E, Vagi S, Fechter-Leggett E, Prudent N, Dott M, et al. Monitoring of Persons with Risk for Exposure to Ebola Virus Disease - United States, November 3, 2014-March 8, 2015. MMWR Morbidity and mortality weekly report. 2015 Jul;64(25):685–9.

12. Centers for Disease Control and Prevention. 2014 Ebola Outbreak in West Africa - Reported Cases Graphs | Ebola Hemorrhagic Fever | CDC [Internet]. Available from: http://www.cdc.gov/vhf/ebola/outbreaks/2014-west-africa/cumulative-cases-graphs.html
